# Investigating microglial modulation of endothelial inflammation through IL1RAP

**DOI:** 10.1002/alz.092771

**Published:** 2025-01-03

**Authors:** Elizabeth S Fisher, Kate Tubbesing, Katherine Stevens, Steven Lotz, Taylor Bertucci, Sally Temple

**Affiliations:** ^1^ Neural Stem Cell Institute, Rensselaer, NY USA

## Abstract

**Background:**

Up to 84% of patients with Alzheimer’s Disease (AD) have vascular damage which precedes cognitive decline. Inflammation induces changes in blood‐brain‐barrier (BBB) integrity, though the link between induction of inflammation and AD is unclear. IL1β, a cytokine upregulated in patients with AD and in mouse models of the disease, is released and interacts with IL1R1 and its obligate co‐receptor, IL1RAP, to induce downstream signaling. A genome wide association study identified a SNP, rs12053868‐G, in *IL1RAP* associated with increased frequency of AD and higher rates of amyloid accumulation. This SNP exerts an independent, additive effect with *APOE44*, suggesting that *IL1RAP* could play a role in modification of AD progression.

**Method:**

*APOE33* or *APOE44* allele‐containing human induced pluripotent stem cells (iPSCs) were differentiated into microglia or purified endothelial cells (ECs), which were validated via RNA sequencing. Differentiated cells were challenged with inflammation, and responses measured. Microglial inflammatory genes were analyzed via qPCR, as well as cytokine release. Barrier resistance of ECs was assessed via ECIS, and they were also evaluated with immunohistochemistry and gene expression changes. To interrogate the role of *IL1RAP* in microglial induction of inflammation, the gene was overexpressed and knocked down in each cell type.

**Result:**

ECs upregulated E‐selectin upon challenge with inflammatory stimuli. ECs also showed alterations in localization of barrier proteins with immunohistochemistry upon inflammatory challenge, and between *APOE33* and *APOE44* cells. Microglia upregulated several cytokines in response to inflammatory challenge.

**Conclusion:**

Human iPSC‐derived microglia and ECs are altered by inflammatory challenge, and *APOE44* ECs show differences in barrier protein localization at rest. Future experiments will investigate the interactions between microglia and ECs in both APOE alleles, as well as their response to overexpression or KD of *IL1RAP*. These experiments will establish the functional role of inflammation in microglia‐EC interactions, and the contributions of these interactions to the development of cerebral vascular damage.

